# Transcriptomic Characterization of Odorant Binding Proteins in *Cacia cretifera thibetana* and Their Association with Different Host Emitted Volatiles

**DOI:** 10.3390/insects12090787

**Published:** 2021-09-03

**Authors:** Ning Zhao, Xiangzhong Mao, Naiyong Liu, Ling Liu, Zhixiao Zhang, Sangzi Ze, Bin Yang

**Affiliations:** 1 Key Laboratory of Forest Disaster Warning and Control of Yunnan Province, Southwest Forestry University, Kunming 650224, China; lijiangzhn@swfu.edu.cn (N.Z.); xlsktw@swfu.edu.cn (X.M.); naiyong.liu@swfu.edu.cn (N.L.); 2 Yunnan Academy of Forestry and Grassland, Kunming 650224, China; liuling_km@126.com (L.L.); zzx_20071988@163.com (Z.Z.); 3 Yunnan Forestry and Grassland Pest Control and Quarantine Bureau, Kunming 650051, China; zesangzi@163.com

**Keywords:** *Cacia cretifera thibetana*, odorant binding proteins, volatiles, repellent, walnut trees

## Abstract

**Simple Summary:**

The odorant binding proteins (OBPs) interact with host chemical compounds to elicit olfactory responses. Transcriptome analysis of six different tissues of male and female *Cacia cretifera thibetana* was performed to unravel the interaction of OBPs with host compounds. In both sexes, differentially expressed genes were associated with the KEGG pathways such as cutin, suberine and wax biosynthesis, glycerophospholipid metabolism, choline metabolism in cancer, and the chemokine signaling pathway. The expression of 11 out of 31 OBPs were confirmed by quantitative RT-PCR and seven were found to be specifically expressed in antennae. CcreOBP6 and CcreOBP10 showed strong affinity for terpineol and trans-2-hexenal exhibiting their potential role as an attractant or repellent to control *C. cretifera thibetana*.

**Abstract:**

This study characterized the transcriptome of *Cacia cretifera thibetana* and explored odorant binding proteins (OBPs) and their interaction with host-specific compounds. A total of 36 samples from six different organs including antennae, head, thorax, abdomen, wings, and legs (12 groups with 3 replicates per group) from both male and female insects were collected for RNA extraction. Transcriptomic analysis revealed a total of 89,897 transcripts as unigenes, with an average length of 1036 bp. Between male and female groups, 31,095 transcripts were identified as differentially expressed genes (DEGs). The KEGG pathway analysis revealed 26 DEGs associated with cutin, suberine, and wax biosynthesis and 70, 48, and 62 were linked to glycerophospholipid metabolism, choline metabolism in cancer, and chemokine signaling pathways, respectively. A total of 31 OBP genes were identified. Among them, the relative expression of 11 OBP genes (OBP6, 10, 12, 14, 17, 20, 22, 26, 28, 30, and 31) was confirmed by quantitative RT-PCR in different tissues. Seven OBP genes including CcreOBP6 and CcreOBP10 revealed antennae-specific expression. Further, we selected two OBPs (CcreOBP6 and CcreOBP10) for functional analysis to evaluate their binding affinity with 20 host odorant compounds. The CcreOBP6 and CcreOBP10 exhibited strong binding affinities with terpineol and trans-2-hexenal revealing their potential as an attractant or repellent for controlling *C. cretifera thibetana*.

## 1. Introduction

The Tibetan longhorn beetle, *Cacia cretifera thibetana* (Coleoptera, Cerambycidae, Lamiinae), causes economic losses as it is the major pest of walnut (*Juglans regia* L.) trees in China. It was firstly discovered in the Yunnan province of China and is now widely distributed across Tibet and other provinces of China i.e., Sichuan, Guangxi, and Yunnan. *C. cretifera thibetana* at different stages of life can harm the plants as the adult beetles mainly consume the bark and leaves of host plant twigs leading to the death of twigs [1], whereas Tibetan longhorn beetles at the larval stage drill the wood of the host plant and eat phloem while cutting off the connection of vascular tissues. It subsequently disrupts the usual nutrient transportation required for normal plant growth and ultimately results in wither growth and serious economic losses including loss of forest trees, fruits, flower, medicinal material, furniture, building wooden material, and so on [2,3].

The olfactory system of insects drives its behaviors that are significantly associated with fitness, such as to locate the appropriate hosts and mates [4]. While searching suitable host material, the beetles usually respond to various host and non-host plant emitted volatiles [5,6,7]. Although, most of the herbivores such as longhorn beetles localize the trees through pheromone aggregation, which were secreted by the beetles that had already attacked the host tree [8]. This signal is responsible for synchronized massive attack, which often results in the death of host tree and extensive forest demolition [4,8]. By employing traps based on plant volatiles and pheromones, the olfactory system of the beetle is the major target for research [8]. Owing to the massive economic and ecological impact of longhorn beetles, comprehensive knowledge about their olfactory physiology and chemical ecology is required [8,9]. In this regard, better understanding of the molecular mechanism of odor detection is imperative to explore the process that drives the beetle’s olfactory physiology and chemical sensation. Such knowledge is a prerequisite to devise potential green pest control strategies by exploring novel repellants or attractants.

The peripheral olfactory proteins possess a crucial functional role in the olfaction process in insects. These include olfactory receptor proteins (ORs), odorant binding proteins (OBPs), ionotropic receptors (IRs), chemosensory proteins (CSPs), and sensory neuron membrane proteins (SNMPs). All of these have involvement at different steps of the sensory signal transduction pathway in insects [10]. The OBPs are small proteins secreted by hydrophilic accessory cells that accumulate in the sensilla lymph [11]. These soluble OBPs enable the transportation of the odorant particles via the sensillar lymph and develop the link between ORs and the external environment [10]. However, limited information is available regarding the molecular mechanisms fundamentally related to olfaction in *C. cretifera thibetana*. Exploring the odorant processing genes repertoire involved in olfaction could offer valuable insights into the chemical mechanisms of insect olfaction that would facilitate the identification of possible chemical targets, which could be manipulated for *C. cretifera thibetana* control.

Presently, removing dead plant branches and the use of chemical insecticides are being adopted as preventive and therapeutic measures to control the attack of *C. cretifera thibetana* [12,13]. However, these measures are not cost-effective and deteriorate the natural fabric of the ecosystem through increasing environmental pollution. In addition, it also develops the pesticide resistance in *C. cretifera thibetana*. Thus, there is a dire need to find green and eco-friendly pest control procedures to replace or reduce the use of harmful chemicals or pesticides. This study comprehensively characterized the transcriptome of *C. cretifera thibetana* for the first time with specific emphasis to find the olfaction-related proteins and their association with host-specific compounds, which can be potentially used as repellents to avoid the herbivore’s attack on walnut plants.

## 2. Materials and Methods

### 2.1. Sample Collection and Preparation

During late May 2018, the injured walnut branches were collected from *Juglans sigillata* in the walnut forest in Midu County, Dali Prefecture, Yunnan Province, China. From mid-June, we observed the emergence of adult *C. cretifera thibetana* daily. The same batch of emerging *C. cretifera thibetana* adults were separated into 50 cm × 50 cm × 50 cm insect cages after identifying them as male and female. For the rearing of insects, the room temperature was 25 ℃, and the relative humidity was maintained at 50–60%. The tissues from different organs including antennae (At), head (H), thorax (T), abdomen (Ab), wings (W), and legs (F) of both female and male adults of *C. cretifera thibetana* were collected on ice and put in a RNase-free centrifuge tube containing Trizol reagent (Table 1). These samples were then stored in the refrigerator at −80 ℃ until further processing.

### 2.2. RNA Extraction and Quantification

A total of 36 samples from six different organs including antennae, head, thorax, abdomen, wings, and legs (12 groups with 3 replicates per group) was collected. RNA was extracted from these tissues using TRizol reagent. RNA degradation and contamination were observed on 1% agarose gels. RNA purity was checked using the NanoPhotometer^®®^ spectrophotometer (IMPLEN, Westlake Village, CA, USA). The concentration of RNA was measured using the Qubit^®®^ RNA Assay Kit in Qubit^®®^ 2.0 Fluorometer (Life Technologies, Westlake Village, CA, USA). RNA integrity was assessed using the RNA Nano 6000 Assay Kit of the Bioanalyzer 2100 system (Agilent Technologies, Westlake Village, CA, USA).

### 2.3. cDNA Library Preparation and Transcriptome Sequencing

For preparations of samples for transcriptome sequencing, a 1.5 μg RNA sample from each tissue was used. The cDNA libraries were prepared using NEBNext^®®^ Ultra™ RNA Library Prep Kit for Illumina^®®^ (NEB, Westlake Village, CA, USA) following the manufacturer’s instructions. First-strand cDNA was synthesized using a random hexamer primer and MMuLV Reverse Transcriptase (RNase H-) followed by second-strand cDNA synthesis using DNA Polymerase I and RNase H. After end repair, A-tailing, and ligation of adapters (NEBNext Adaptor), the products were amplified with Phusion High-Fidelity DNA polymerase, Universal PCR primers, and Index (X) primer was performed. In order to select cDNA fragments preferentially 150~200 bp in length, the library fragments were purified with the AMPure XP system (Beckman Coulter, Beverly, CA, USA). Finally, the PCR products were purified (AMPure XP system) and library quality was assessed on the Agilent Bioanalyzer 2100 system. The cDNA libraries were sequenced on the Illumina HiSeq2000 platform and paired-end reads were obtained.

### 2.4. Quality Control, De Novo Assembly and Functional Annotation

All the raw reads were firstly processed through in-house perl scripts to remove low-quality sequences and reads containing an adapter, and ploy-N. The quality control criteria included: (i) Removal of the thread with adapter; (ii) removal of unidentified bases (N) ratio greater than 10% of the reads; (iii) removal of low-quality reads (the number of bases with a mass Q ≤ 20 accounting for more than 50% of reads). All downstream analyses were performed on reads that passed quality control. The clean reads were assembled using De novo transcriptome assembly with the Trinity (Version: r2013-11-10) using default parameters [14]. The assembled transcripts were hierarchically clustered to unigenes using shared reads and expression by Corset [15]. The annotation of unigenes was performed by searches against the Nr protein database (http://www.ncbi.nlm.nih.gov/, accessed on 20 March 2021), Swiss Prot (http://www.expasy.ch/sprot/, accessed on 20 March 2021), Gene Ontology (GO) (http://www.geneontology.org, accessed on 20 March 2021), Kyoto Encyclopedia of Genes and Genomes (KEGG) (http://www.genome.jp/kegg/, accessed on 20 March 2021), and eggnog (http://eggnogdb.embl.de/, accessed on 20 March 2021) databases with an E-value threshold of 1 × 10^−5^.

### 2.5. Identification of Odor Binding Protein (OBP) Genes

With tBLASTn, the available sequences of OBPs from Insecta species were used as queries to identify candidate unigenes [5,6]. All candidate OBPs were manually checked by assessing the NCBI BLASTx results [7].

### 2.6. Quantitative Real-Time PCR

Quantitative real-time PCR (qRT-PCR) was performed to evaluate the expression of candidate OBP genes. The total RNA from different organs (antennae, head, thorax, abdomen, wings, and legs) was extracted as described above. The cDNA synthesis was performed using the PrimeScript RT Reagent Kit with gDNA Eraser (No. RR047A; TaKaRa, Shiga, Japan). Gene-specific primers were designed using Primer3 (http://bioinfo.ut.ee/primer3-0.4.0/, accessed on 20 March 2021). For qRT-PCR, SYBR Premix Ex Taq™ II (No. RR820A; TaKaRa) was used in the Bio-Rad CFX96 PCR System (Hercules, CA, USA). The β-actin gene as a reference gene was selected from the transcriptome of *C. cretifera thibetana*. For each tissue sample, a set of three biological and three technical replicates was employed. Two qRT-PCR amplification conditions were used for expression profiling of 11 genes including OBP6, OBP10, OBP12, OBP14, OBP17, OBP20, OBP22, OBP26, OBP28, OBP30, and OBP31 in different tissues including abdomen (Ab), antennae (At), head (H), thorax (C), legs (F), and wings (W) of *C. cretifera thibetana* female and male adults. First, for the amplification curve, reaction conditions were 95 ℃ for 30 s, followed by 40 cycles of 95 ℃ for 5 s, 60 ℃ for 20 s, and 72 ℃ for single-point detection signal. Second, the reaction conditions for dissociation curve: 95 ℃ for 0 s, 65 ℃ for 15 s, 95 ℃ for 0 s, and continued detection of the signal. Bio-Rad CFX Manager (version 3.1.1517.0823) was used to normalize expression based on ΔΔCq values, and the 2−ΔΔCT method was used (the amplification efficiency for 11 genes was equal to 100%).

### 2.7. Differential Expression Analysis

Differential expression analysis among male and female groups was performed using the DESeq R package (1.18.0). The resulting *p*-values were adjusted using Benjamini and Hochberg’s approach for controlling the false discovery rate. Genes with an adjusted *p* value < 0.05 were considered as differentially expressed.

### 2.8. GO and KEGG Enrichment Analysis of Differentially Expressed Genes

Gene Ontology (GO) enrichment analysis of differentially expressed genes was performed in R (GOseq R Package) using corrected gene length bias. The GO terms with corrected *p* value < 0.05 were considered significantly enriched by DEGs. KOBAS software was used to test the statistical enrichment of DEGs in KEGG pathways (http://www.genome.jp/kegg/, accessed on 20 March 2021).

### 2.9. Evaluation of Binding Affinities of OBP Genes with Host Compounds

The binding affinities of both CcreOBP6 and CcreOBP10 proteins with 20 odor volatiles were determined. The CcreOBP6 and CcreOBP10 proteins from prokaryotic cells were purified by Ni affinity chromatography. Then, 50 mM Tris-HCL buffer solution (pH = 7.4) was prepared, and N-phenyl-1-naphthylamine (1-NPN) was used as a fluorescent probe. For chromatography, the methanol was used as a solvent for fluorescent probe 1-NPN as well as for 20 odor standard volatile samples dissolved in solvent to make a 1 mM solution. These samples were added to a 96 Micro Well TM microtiter plate (NunclonTM) while the multifunctional microplate reader (VARIOSKAN FLASH) excitation light wavelength was set to 337 nm. The scanning wavelength was between 370 to 550 nm, and both excitation and emission slits were set to 5 nm. To determine the CcreOBP6 and CcreOBP10 protein binding constant with the fluorescent probe 1-NPN, a solution was prepared with CcreOBP6 and CcreOBP10 proteins in Tris-HCL (pH = 7.4) buffer with a 2 μM final concentration. The successive concentrations of 2, 4, 6, 8, 12, 16, and 20 μM of 1-NPN were added, and the fluorescence intensity was recorded at the maximum emission wavelength (Em = 410 nm) for each time, and the experiment was repeated three times. The formula for the protein and odor standard dissociation constant was calculated by: Ki = [IC 50]/(1 + [1-NPN]/K 1-NPN) where IC 50 denotes the odor standard concentrations as the fluorescence intensity value was decreased by half, [1-NPN] is the unbound 1-NPN prob concentration, and K 1-NPN is the protein and 1-NPN probe binding constant.

## 3. Results

### 3.1. The RNA-Sequencing Data

**Figure 1:**
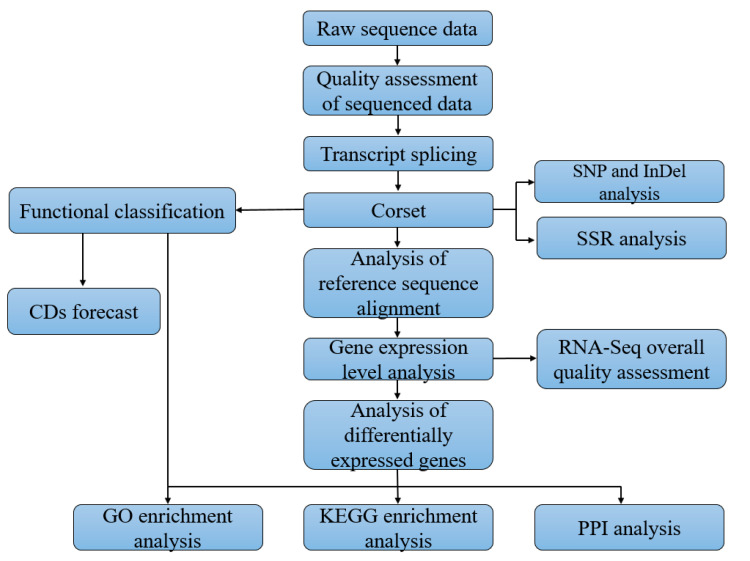
The workflow diagram of RNA-seq data analysis.

Illumina HiseqTM high-throughput sequencing was used to obtain the raw data files of all the samples, and Casava Base Calling analysis was used to transfer the raw data into sequence reads, stored in FASTQ (FQ) file format. The Illumina sequencing identifiers of the FQ file are described in Table S1 and the overall workflow diagram of RNA-seq data analysis is presented in Figure 1. 

### 3.2. De Novo Assembly and Transcriptome Annotation of C. cretifera thibetana

From all samples of the two groups, the identified raw reads ranged 19,720,506–28,403,775 (Table S2). After the removal of adapter sequences, reads with N > 10% were considered low-quality reads (QPHRED ≤ 20), and considering reads with a base percentage more than 50%, a total of 19,691,465–28,355,944 clean reads were screened out with a 2.95 G to 4.25 G clean bases ratio. Moreover, 99% of bases from each sample were correctly determined with a 0.02% error rate (Table S2), while the percentage of GC contents ranged from 39.92 to 42.85% (Table S2).

**Figure 2:**
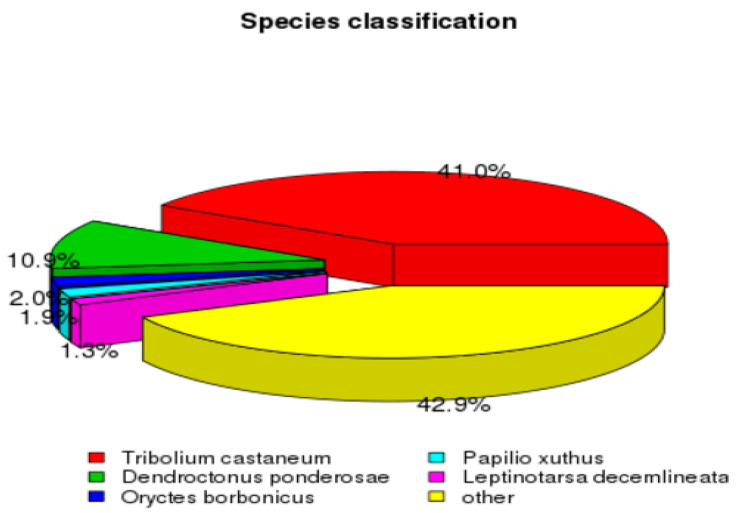
The unigene BLASTx searches against the Nr database for species distribution analysis.

Three modules of Trinity software were used independently to process RNA-seq data and the obtained clean reads were spliced to develop the reference sequences for successive analyses. CORSET hierarchical clustering was used to obtain the longest Cluster sequence and the transcripts along with the calculation of their corresponding length (Tables S3 and S4). A total of 222,946 transcripts were generated with a mean length of 807 bp (with a range of 201 to 31,659 bp). Of these transcripts, 89,897 were assigned the status of unigene, with an average length of 1036 bp (Tables S3 and S4). Moreover, similarity searches were performed against seven different databases for functional annotation of the assembled unigenes. As a result, 33,735 (37.52%), 14,083 (15.66%), 14,694 (16.34%), 25,481 (28.34%), 28,563 (31.77%), 29,374 (32.67%), and 13,940 (15.5%) unigenes were matched to NR, NT, KO, SwissProt, PFAM, GO, and KEGG database, respectively, whereas 5256 (5.84%) unigenes were annotated in all databases and 41,225 (45.85%) were annotated in at least one database. The unigenes BLASTx search against the Nr database for species distribution exhibited a higher percentage of similarity of about 41% with *Tribolium castaneum* followed by *Denedroctonus ponderosae* (10.9%), *Oryctes borbonicus* (2%), *Papilio xuthus* (1.9%), and *Leptinotarsa decemlineata* (1.3%) (Figure 2). 

### 3.3. The Unigene Functional Annotation

**Figure 3:**
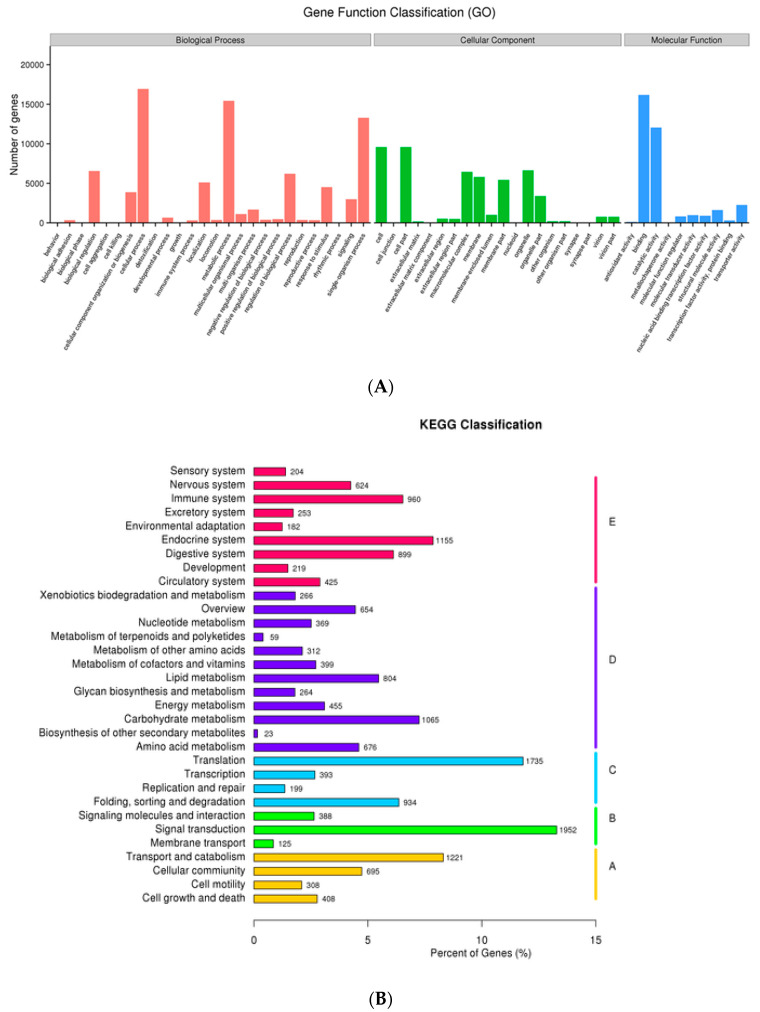
(**A**) The unigene GO annotation including the biological process, cellular component, and molecular function. (**B**) The KEGG classification of unigenes, where A is Cellular process, B is Environmental information processing, C is Genetic Information Processing, D is Metabolism, and E is Organismal Systems.

For unigenes, the GO clustering annotated 26 biological process (BP) groups, which were sorted for 20 different cellular components (CC) that further have their functional involvement in 10 molecular function (MF) types. ‘Cellular process’, ‘metabolic process’, and ‘single-organism process’ were the abundant terms related to BP where >45,000 unigenes were involved, while there were ‘cell’, ‘cell part’, ‘macromolecule complex’, and ‘organelle’ in the CC group (>40,000) and ‘binding’ and ‘catalytic activities’ (>27,000) in the MF categories (Figure 3A). Furthermore, KEGG enrichment analysis categorized 17,585 (19.56%) unigenes into five KEGG classification types, which were further characterized into 32 subgroups. The frequency of the unigenes in five KEGG groups were ordered as: ‘organism system’ (3881; 22.06%), ‘metabolism’ (5346; 30.4%), ‘genetic information processing’ (3261; 18.54%) ‘environmental information processing’ (2465; 14.01%), and ‘cellular process’ (2632; 14.96%). Additionally, signal transduction (1952), translation (1735), transport and catabolism (1221), the endocrine system (1155), and carbohydrate metabolism (1065) were the predominant subgroups (Figure 3B).

### 3.4. Gene Expression Analyses

#### 3.4.1. The RNA-Seq Data Mapping to Reference Sequence and Transcripts Distribution in Samples

**Figure 4:**
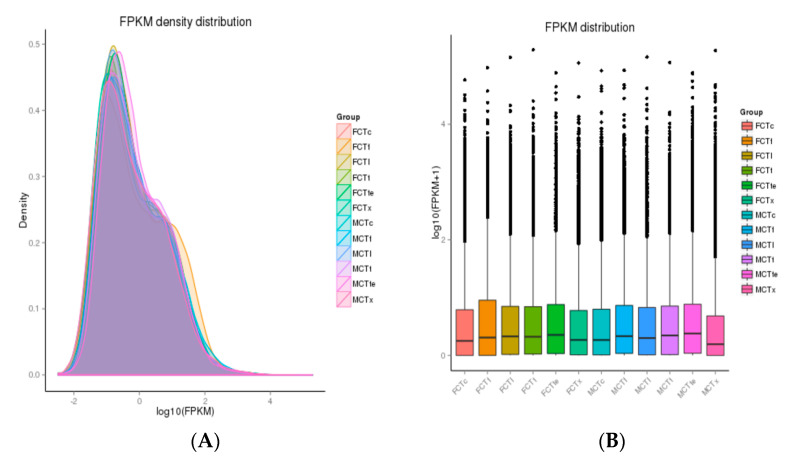
(**A**) The transcript FPKM density for each of the female and male samples. (**B**) The box chart presentation of different female and male sample expression levels.

The total reads data for both group samples ranged between 39 and 56 million and the reference sequence developed by Trinity was further used to map the clean data reads. For both groups, the mapped percentage (%) alignment to the reference sequence ranged between 77.23 and 83.74% (Table S5). For each sample type, the relative abundance of transcripts in terms of fragments per kilobase per million mapped reads (FPKM) was measured to normalize the RNA-seq data. The transcript FPKM density for each sample is presented in Figure 4A, whereas the expression levels in the different samples are shown in Figure 4B as a boxplot chart.

#### 3.4.2. Identification of Differentially Expressed Genes and Functional Enrichment Analyses

**Figure 5:**
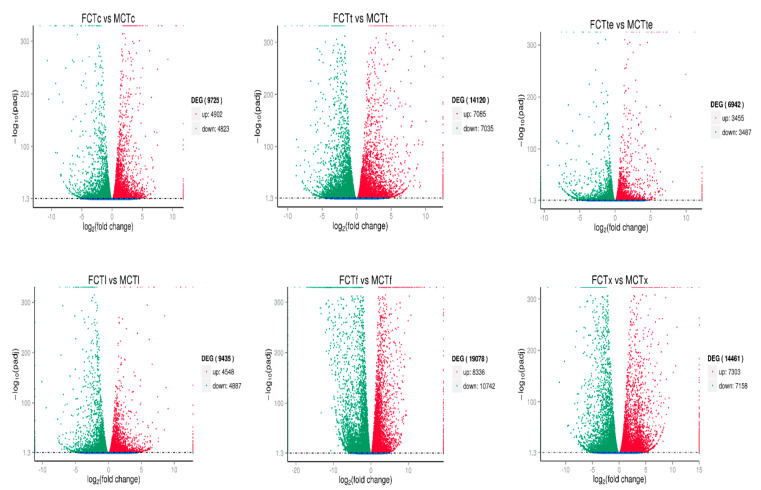
Differential gene expression analysis of two group samples (female vs. male).

To screen the DEGs, we used fold change value >2 and *p* < 0.05 as a criterion. A total of 89,897 transcripts were obtained from RNA-seq data, out of which 31,095 were identified as DEGs. Further, the DEGs of each sample from two groups (female vs. male) were compared (Figure 5). The ratio of up- and downregulated genes in different tissues of both groups (female vs. male) was 4902:4823 (FCTc vs. MCTc), 7085:7035 (FCTt vs. MCTt), 3455:3487 (FCTte vs. MCTte), 4548:4887 (FCTI vs. MCTI), 8336:10742 (FCTf vs. MCTf), and 7303:7158 (FCTx vs. MCTx) (Figure 5). 

Moreover, GO enrichment analysis was used to explore the functions of DEGs in two groups, and abundant GO terms along with their classification are shown in Figure S1A–F. The top terms of GO enrichment analysis, biological process and molecular function, with the number of DEGs and GO accession are presented in Table 2. It was observed that 730 DEGs out of 6448 exhibited a significant association with the oxidation reduction process. Meanwhile, 702, 145, and 156 DEGs were involved in molecular function including oxidoreductase activity acting on paired donors, oxidation or reduction of molecular oxygen, and heme binding, respectively (Table 2).

Additionally, the KEGG pathway analysis is helpful to explicate the significant enriched pathways for DEGs through which these genes perform their biological functions. The statistics of the top 20 KEGG enrichment pathways with the number of the genes for both groups are presented in Figure S2A–F, while the DEG number with pathway ID and KEGG enrichment term is shown in Table 3. Roughly 26 DEGs out of 40 were found to be associated with cutin, suberine, and wax biosynthesis, whereas 70 out of 152, 48 out of 98, and 62 out of 134 were associated with glycerophospholipid metabolism, choline metabolism in cancer, and chemokine signaling pathway, respectively, as seen in Table 3.

#### 3.4.3. Quantitative Real-Time PCR Analysis

**Figure 6:**
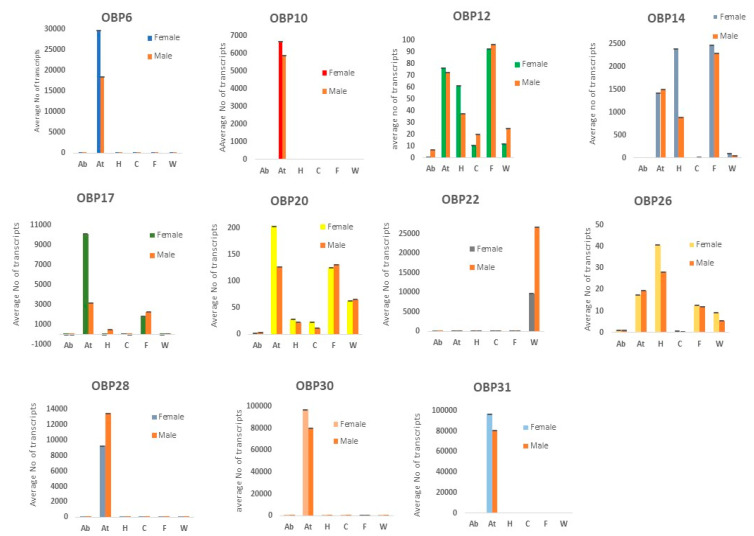
Relative expression (average number of transcripts) of 11 OBP genes in different tissues. Relative fold changes were normalized to transcript levels in the abdomen tissue. (Ab = abdomen; At = Antennae; H = Head; C = thorax; F = legs; W = wing).

A total of 31 OBPs were identified and the qRT-PCR of 11 OBP genes were performed to evaluate their expression in different tissues. The results revealed that 7 genes (OBP6, 10, 17, 20, 28, 30, and 31) out of 11 showed substantially higher expression in antenna as compared to other tissues. Two genes, OBP12 and 14, showed the highest expression in legs while *OBP22* showed higher expression in wing tissues. The OBP26 showed highest expression in head followed by antenna tissue (Figure 6). All these genes showed comparatively higher expression in respective tissues of females as compared to males except in the case of *OBP22* and *28,* which revealed higher expression in males. Based on these results, we selected *OBP6* and *OBP10* for further analysis for the investigation of binding affinities with volatile compounds, from those genes that were exclusively expressed in antenna tissues and exhibited fair expression in both sexes. 

#### 3.4.4. Functional Analysis of Odorant Binding Proteins

**Figure 7:**
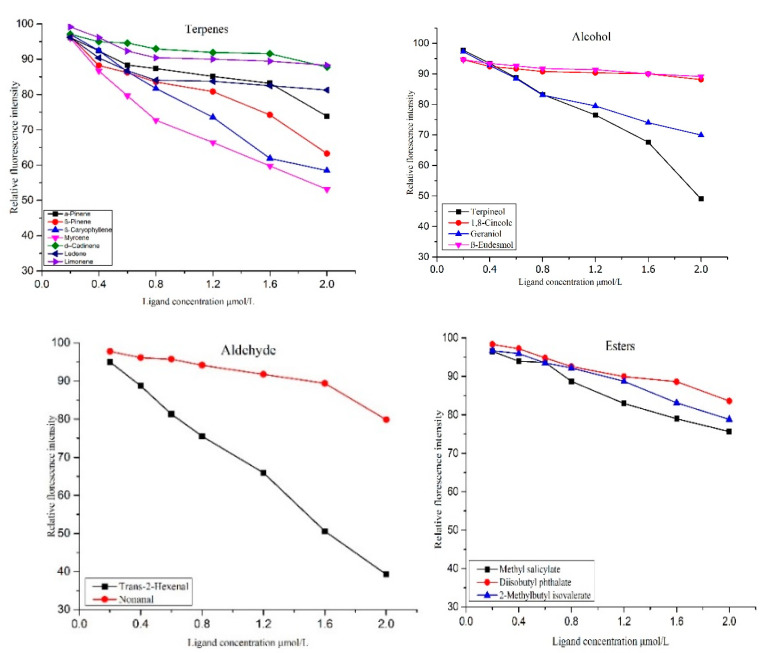
Competitive binding curves of CcreOBP6 to different ligands including terpenes, aldehyde, alcohol, and esters.

Furthermore, for genes with a significant role in host plant protection, we identified a total of 31 OBP genes of which CcreOBP6 and CcreOBP10 were further analyzed for binding affinities with 20 host-secreted small molecules including nine terpenes, five alcohols, three ketones, and three aldehydes. The CcreOBP6, CcreOBP10, and fluorescent probe 1-NPN exhibited higher binding affinities, although the dissociation constant for CcreOBP6 and CcreOBP10 were observed as 7.09 and 6.18 μmol/L, respectively (Figure S3). Of the selected 20 host odorant compounds, the CcreOBP6 showed binding capabilities with only 16 molecules, where the relative fluorescence values for these compounds were below 50% (Figure 7). Additionally, CcreOBP6 did not show any binding with caryophyllene oxide, 1,6-cyclodecadiene, n-hexanal, and phytol (Figure 7). The IC50 and Ki values for terpineol and trans-2-hexenal were 19.71 and 16.25, and 15.64 μmol/L and 12.90 μmol/L, respectively, which revealed the strongest binding ability of trans-2-hexenal with CcreOBP6. The eucalyptol showed the weakest binding affinity as the relative fluorescence value was only 10.84% (Figure 7 and Table S6).

**Figure 8:**
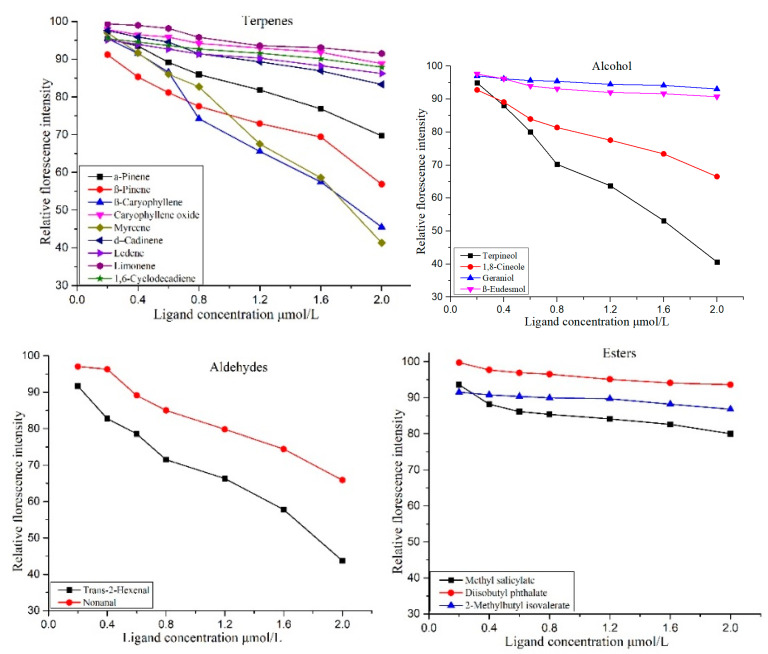
Competitive binding curves of CcreOBP10 to different ligands including terpenes, aldehyde, alcohol, and esters.

Moreover, the CcreOBP10 showed binding with 18 host chemical compounds including n-hexanal and phytol, which showed no binding affinities with CcreOBP6 (Figure 7 and Figure 8). Myrcene, 1-caryophyllene, terpineol, and trans-2-hexenal were the odor compounds with a relative fluorescence value below 50%. Out of these 18 molecules, the CcreOBP10 exhibited the strongest binding affinity to terpineol with an IC50 value of 16.54 and a Ki value of 12.73 μmol/L, followed by myrcene with an IC 50 value 17.91 and a Ki value of 13.81 μmol/L. The lowest binding affinity was revealed as di-isobutyl phthalate, as only a 6.4% drop in the relative fluorescence value was observed (Figure 8 and Table S6).

Both the CcreOBP6 and CcreOBP10 genes showed strong binding affinities with terpineol and trans-2-hexenal, which revealed that the *C. cretifera thibetana* can sense these host-secreted odorant compounds. This reveals their potential role in the metabotropic signaling pathway in these insect species. Furthermore, the KEGG pathway analysis of CcreOBP6 and CcreOBP10 exhibited that the binding of odor compounds (as ligand) with CcreOBP6 and CcreOBP10 receptor proteins could increase the intracellular level of cAMP by activating type III adenylyl cyclase after coupling with olfactory specific Gs-protein (G). This cAMP targets the olfactory-specific ionic channel gates allowing the downwards movement of the cation, including Ca and Na, into the cell from their electrochemical gradients and depolarizes the olfactory receptor neurons (ORNs). Moreover, the Ca flow into the cell results in the activation of a Ca-activated Cl channel, further increasing the depolarization by permitting the outward movement of Cl from the cell.

## 4. Discussion

In insects, the olfactory system is crucial for survival and reproduction [11,15,16,17,18,19,20,21]. Insect olfactory sensors perceive odorant messages including host or non-host volatiles and pheromones, and subsequently initiate the physiological signals that eventually influence the behavior of insects [16]. This study aimed to comprehensively characterize the transcriptome of *C. cretifera thibetana* with specific emphasis to explore the OBPs and their ability to bind with host-specific chemical compounds, which can potentially be used as a repellent or attractant to avoid the herbivore’s attack on walnut plants.

*Cacia cretifera thibetana* belongs to the most species-rich long-horned beetle family Cerambycidae with more than 26,000 described species including a significant number of pests around the globe [22,23]. However, it remained underrepresented in terms of available genome and transcriptome resources. We have generated a comprehensive transcriptome sequence resource for *C. cretifera thibetana* targeting the wings, legs, abdomen, thorax, head, and antennae tissues, which yielded 222,946 transcripts, out of which 89,897 were assigned the status of unigene and exhibited a higher percentage of similarity (about 41%) with *Tribolium castaneum*. The transcriptome resources developed in this study are complementary to other available transcriptome resources of bark beetles including pine beetles [24,25,26] and coffee berry borer [27].

In insects, the well-equipped antennae with wide ranging small sensory hair structures (sensilla) are crucial for the olfactory system. The dendrites of ORNs protrude into the antennal lymph and facilitate the peripheral olfactory signal transduction [28]. The ORNs are characterized as a biological transducer that convert environment-related volatile signals into a sensual input [16]. The whole olfactory system relies on the receptor types that are expressed on peripheral ORNs [11]. Further, the insect’s peripheral system has the ability for selective detection of minor quantities of odorant and process the information to the central nervous system [11]. 

A variety of olfaction-associated proteins, including the OBPs, have also been reported as the core proteins with a significant role in the odor-sensation process. These proteins are also involved in the transportation of odorant molecules through the sensillum lymph and serve as a link between ORs and the external environment [28]. A total of 31 OBP genes were identified in the present study through antennal transcriptomic analysis of *C. cretifera thibetana*. The OBPs belong to a very diverse groups of proteins in insects, and the number of coding genes for these proteins range from 13 in some ant species [29] to more than 100 in several mosquitoes [30]. Earlier studies on the antennal transcriptome of the bark beetle have reported 21 OBP transcripts [31]. Similarly, three earlier studies reported 42 OBPs in the longhorn beetle [8,32,33]. Moreover, Liu et al. [28] reported 31 putative OBPs in the bark beetle (*D. ponderosa*).This diverse nature of OBPs in different insect species indicate the need for their functional versatility to sense diverse host molecules found abundantly in nature. Moreover, these OBPs are expressed in most organs of the insect body and also have non-conventional roles including in taste, immunity response, and humidity detection [34].

Our findings revealed that seven OBP genes were exclusively expressed in the antenna tissue only (antennae enriched expression) as it is the main tissue involved in the olfactory sensation process. These findings are in agreement with earlier studies revealing that insect antennae-specific OBPs are crucially important for odorant recognition and sensitivity in insects [35]. It is well established that antennae-enriched expression of OBPs is reflective of their active role in olfactory perception in insects [36]. Antennae-specific OBPs are intimately involved in the olfaction process, and mediate the detection of pheromones and/or volatile compounds of the host plant [37]. The OBPs are primary proteins that are essentially required for olfactory behavior and activity of pheromone-sensitive neurons as they activate the receptor complex to respond to odorant molecules [38]. These characteristics make OBPs potential candidates for insect pest management (IPM) and offer a horizon of opportunities to devise potential environmentally friendly pest control strategies to replace chemical insecticides. Moreover, out of 11 OBP genes, 9 showed comparatively higher expression in females compared to males, except OBP22 and 26, which were highly expressed in males. This indicates the possibility of using these OBP genes for developing sex-specific attractants or repellents but requires further in vivo studies to corroborate these findings. 

In the present study, we selected 20 natural host-plant-secreted volatiles to screen their properties as an insect attractant or repellent by evaluating their binding affinities with OPB6 and OBP10 through competitive binding assay. We selected these two OBPs based on their antennae-enriched expression owing to their involvement in olfactory sensation as described above. For the CcreOBP6 and CcreOBP10 genes of *C. cretifera thibetana*, terpineol and trans-2-hexenal exhibited strong binding affinities, which revealed that these insects can sense these odorant compounds. This potential binding ability of these compounds indicates their potential role in the metabotropic signaling pathway and depolarization of the neuron by affecting the ionic channels [39,40]. Chen et al. reported a significant repellent behavioral response of trans-2-hexenal for males and females by using an olfactometer and demonstrated that a lower dose of trans-2-hexenal had good repellent activity [41]. A recent study has shown the potential increase in the concentration of phytochemicals including hexenal along with other alkaloids, terpenes, esters, and aldehydes in walnut under stress conditions [42]. It indicates the significance of these compounds for the health and survival of host plants and fruit quality. Moreover, earlier studies [38,39] have also reported that terpineol enhances the olfactory responses and produces a sensible repellent effect against the insects. The findings of the present study strongly indicate the potential of terpineol and trans-2-hexenal as repellent or attractant volatile host secreted compounds, which could be used as a green and efficient measure to control *C. cretifera thibetana.*

## 5. Conclusions

Transcriptomic analysis of *C. cretifera thibetana* identified 31 OBP genes. Out of the selected 11 OBP genes, 7 OBP genes showed higher relative expression in antennae tissue revealing their potential role in olfaction. The CcreOBP6 and CcreOBP10 exhibited strong binding affinities with terpineol and trans-2-hexenal, which revealed that *C. cretifera thibetana* can sense these host-secreted odorant compounds as they depolarize the neurons and influence the metabotropic signaling pathway. The findings of the present study strongly indicate the potential of terpineol and trans-2-hexenal as repellent or attractant volatile host compounds, which could be used as a green and efficient measure to control *C. cretifera thibetana*. Further molecular and functional studies of these OBP genes are essentially required to explore their potential as novel targets for devising potential ecofriendly measures of controlling *C. cretifera thibetana*.

## Figures and Tables

**Table 1:** Details of samples collected from different organs.

Sr. No	Female Samples		Male Samples	
	Organ/Tissue	Abbreviations	Organ/Tissue	Abbreviations
1	Female wings	FCTc	Male wings	MCTc
2	Female abdomen	FCTf	Male Abdomen	MCTf
3	Female legs	FCTl	Male legs	MCT1
4	Female head	FCTt	Male head	MCTt
5	Female antennae	FCTte	Male antennae	MCTte
6	Female thorax	FCTx	Male thorax	MCTx

**Table 2:** The top terms of GO enrichment analysis.

GO Accession	Description	Term Type	Over Represented *p*-Value	Corrected *p*-Value	DEG Item	DEG List
GO:0055114	oxidation reduction process	Biological Process	1.2268 × 10^−15^	8.0529 × 10^−12^	730	6448
GO:0016491	Oxidoreductase activity	Molecular Function	1.1892 × 10^−14^	3.903 × 10^−11^	702	6448
GO:0016705	oxidoreductase activity, acting on paired donors, oxidation or reduction of molecular oxygen	Molecular Function	1.7425 × 10^−13^	3.8127 × 10^−10^	145	6448
GO:0020037	heme binding	Molecular Function	3.234 × 10^−13^	5.307 × 10^−10^	156	6448

**Table 3:** KEGG significant enrichment analysis of DEGs.

Term	ID	Sample Number	Background Number	*p*-Value
Cutin, suberine and wax biosynthesis	ko00073	26	40	0.0046
Glycerophospholipid metabolism	ko00564	70	152	0.0086
Choline metabolism in cancer	ko05231	48	98	0.0119
Chemokine signaling pathway	ko04062	62	134	0.0119

## Data Availability

The raw transcriptome data have been submitted to NCBI SRA under the project number: PRJNA728504.

## Supplementary Materials

The following are available online at https://www.mdpi.com/article/10.3390/insects12090787/s1, Figure S1: GO enrichment analysis of each sample between two groups (female vs. male), Figure S2: The statistics of 20 top KEGG pathways of DEGs, Figure S3: The binding curves of (A) of CcreOBP6 and 1-NPN (B) CcreOBP10 and 1-NPN. Table S1: The details of Illumina sequencing identifiers, Table S2: The quality assessment of sample sequencing output data, Table S3: List of splicing length and frequency distribution, Table S4: List of splicing length distribution, Table S5: The statistics of clean reads mapped to reference sequence.
