# Supplementary material for: Transcriptomic Characterization of Odorant Binding Proteins in Cacia cretifera thibetana and Their Association with Different Host Emitted Volatiles
Source: Insects. 2021 Sep 3;12(9):787. doi: 10.3390/insects12090787 (PMC8469897; doi:10.3390/insects12090787)
Supplement: Supplementary file 1 [file insects-12-00787-s001.zip › insects-1327822-supplementary.pdf]

Supplementary Materials

Table S1. The detail of Illumina sequencing identifiers.

Characteristic	Implication/meaning/connotation/significance
HWI-ST1276	Instrument – unique identifier of the sequencer
71	Run number – Run number on instrument
C1162ACXX	FlowCell ID – ID of flowcell
1	LaneNumber – positive integer
1101	TileNumber – positive integer
1208	X – x coordinate of the spot. Integer which can be negative
2458	Y – y coordinate of the spot. Integer which can be negative
1	ReadNumber - 1 for single reads; 1 or 2 for paired ends
N	whether it is filtered - NB：Y if the read is filtered out, not in the delivered fastq file, N otherwise
0	control number - 0 when none of the control bits are on, otherwise it is an even number
CGATGT	Illumina index sequences

Table S2. The quality assessment of sample sequencing output data.

Sample	Raw Reads	Clean reads	Clean bases	Error (%)	Q20 (%)	Q30 (%)	GC (%)
FCTc1_1	26558303	26523041	3.98G	0.02	98.07	94.31	42.77
FCTc1_2	26558303	26523041	3.98G	0.02	97.68	93.12	42.78
FCTc2_1	27551479	27498088	4.12G	0.02	98.05	94.27	42.68
FCTc2_2	27551479	27498088	4.12G	0.02	96.51	90.46	42.67
FCTc3_1	22226428	22191360	3.33G	0.02	98.09	94.33	42.85
FCTc3_2	22226428	22191360	3.33G	0.02	97.19	91.92	42.85
FCTf1_1	23940811	23898734	3.58G	0.02	98.07	94.28	39.94
FCTf1_2	23940811	23898734	3.58G	0.02	96.29	89.93	39.93
FCTf2_1	20924895	20887135	3.13G	0.02	98.04	94.23	39.93
FCTf2_2	20924895	20887135	3.13G	0.02	96.29	89.89	39.92
FCTf3_1	23336291	23307806	3.5G	0.02	98.10	94.35	40.40
FCTf3_2	23336291	23307806	3.5G	0.02	97.04	91.56	40.40
FCTl1_1	26321827	26282207	3.94G	0.02	98.02	94.18	41.45
FCTl1_2	26321827	26282207	3.94G	0.02	97.56	92.83	41.46
FCTl2_1	23076679	23031905	3.45G	0.02	98.05	94.27	41.36
FCTl2_2	23076679	23031905	3.45G	0.02	96.48	90.39	41.35
FCTl3_1	21139188	21102875	3.17G	0.02	98.04	94.27	41.89

FCTl3_2	21139188	21102875	3.17G	0.02	97.37	92.42	41.89
FCTt1_1	24972375	24937356	3.74G	0.02	98.09	94.34	40.57
FCTt1_2	24972375	24937356	3.74G	0.02	97.31	92.18	40.55
FCTt2_1	27363718	27303692	4.1G	0.02	98.12	94.38	40.12
FCTt2_2	27363718	27303692	4.1G	0.02	96.36	90.03	40.12
FCTt3_1	26427448	26386697	3.96G	0.02	98.11	94.39	40.41
FCTt3_2	26427448	26386697	3.96G	0.02	97.27	92.09	40.40
FCTte1_1	23431685	23399785	3.51G	0.02	98.07	94.32	42.33
FCTte1_2	23431685	23399785	3.51G	0.02	97.53	92.75	42.33
FCTte2_1	22569797	22541127	3.38G	0.02	98.02	94.16	42.39
FCTte2_2	22569797	22541127	3.38G	0.02	97.45	92.55	42.38
FCTte3_1	23928687	23898642	3.58G	0.02	98.00	94.10	42.64
FCTte3_2	23928687	23898642	3.58G	0.02	97.23	92.07	42.63
FCTx1_1	27005073	26966062	4.04G	0.02	98.09	94.31	41.82
FCTx1_2	27005073	26966062	4.04G	0.02	97.25	92.06	41.81
FCTx2_1	28403775	28355944	4.25G	0.02	98.11	94.38	41.68
FCTx2_2	28403775	28355944	4.25G	0.02	96.95	91.35	41.68
FCTx3_1	27825275	27769672	4.17G	0.02	98.08	94.34	41.60
FCTx3_2	27825275	27769672	4.17G	0.02	96.67	90.77	41.58
MCTc1_1	22651416	22620452	3.39G	0.02	98.07	94.28	42.39
MCTc1_2	22651416	22620452	3.39G	0.02	97.20	91.97	42.39
MCTc2_1	22169055	22135227	3.32G	0.02	98.01	94.13	42.57
MCTc2_2	22169055	22135227	3.32G	0.02	97.12	91.82	42.58
MCTc3_1	21707611	21676292	3.25G	0.02	98.09	94.33	42.39
MCTc3_2	21707611	21676292	3.25G	0.02	97.64	92.98	42.40
MCTf1_1	19720506	19691465	2.95G	0.02	97.36	93.98	41.37
MCTf1_2	19720506	19691465	2.95G	0.02	97.15	91.82	41.39
MCTf2_1	24934809	24899740	3.73G	0.02	98.01	94.10	42.24
MCTf2_2	24934809	24899740	3.73G	0.02	96.67	90.77	42.25
MCTf3_1	26849757	26804131	4.02G	0.02	98.05	94.22	41.67
MCTf3_2	26849757	26804131	4.02G	0.02	97.01	91.53	41.68
MCTl1_1	20907708	20879561	3.13G	0.02	98.03	94.19	41.29
MCTl1_2	20907708	20879561	3.13G	0.02	97.53	92.76	41.29
MCTl2_1	26369483	26334432	3.95G	0.02	98.03	94.16	41.64
MCTl2_2	26369483	26334432	3.95G	0.02	97.35	92.30	41.66
MCTl3_1	20946231	20912929	3.14G	0.02	98.08	94.32	41.43
MCTl3_2	20946231	20912929	3.14G	0.02	97.39	92.45	41.42
MCTt1_1	24656373	24557000	3.68G	0.02	98.06	94.20	42.13
MCTt1_2	24656373	24557000	3.68G	0.03	93.24	83.85	42.16

MCTt2_1	21458893	21422372	3.21G	0.02	98.02	94.20	42.23
MCTt2_2	21458893	21422372	3.21G	0.02	97.42	92.58	42.24
MCTt3_1	24834542	24782914	3.72G	0.02	97.99	94.14	42.23
MCTt3_2	24834542	24782914	3.72G	0.02	96.68	90.86	42.23
MCTte1_1	22118594	22076801	3.31G	0.02	97.96	94.11	42.41
MCTte1_2	22118594	22076801	3.31G	0.02	96.98	91.60	42.39
MCTte2_1	25766171	25721281	3.86G	0.02	97.96	94.15	42.64
MCTte2_2	25766171	25721281	3.86G	0.02	97.00	91.69	42.63
MCTte3_1	22657015	22612197	3.39G	0.02	97.95	94.08	42.42
MCTte3_2	22657015	22612197	3.39G	0.02	96.42	90.34	42.43
MCTx1_1	28331802	28283785	4.24G	0.02	98.15	94.45	41.70
MCTx1_2	28331802	28283785	4.24G	0.02	96.52	90.39	41.67
MCTx2_1	26023475	25966498	3.89G	0.02	98.06	94.30	41.81
MCTx2_2	26023475	25966498	3.89G	0.02	96.18	89.75	41.79
MCTx3_1	26247767	26206430	3.93G	0.02	98.10	94.31	41.67
MCTx3_2	26247767	26206430	3.93G	0.02	96.60	90.55	41.64

Table S3. List of splicing length and frequency distribution.

Transcript length interval	200-500bp	500-1kbp	1k-2kbp	>2kbp	Total
Number of transcripts	137107	38224	25856	21759	222946
Number of Genes	34756	28353	15423	11365	89897

Table S4. List of splicing length distribution.

	Min Length	Mean Length	Median Length	Max Length	N50	N90	Total Nucleotides
Transcripts	201	807	374	31659	1584	280	179842879
Genes	201	1036	614	31659	1626	446	93149344

Table S5. The statistics of clean reads mapped to reference sequence.

Sample name	Total reads	Total mapped
FCTc1	53046082	43022214(81.10%)
FCTc2	54996176	44277360(80.51%)
FCTc3	44382720	35987122(81.08%)
FCTf1	47797468	38072994(79.65%)
FCTf2	41774270	33311418(79.74%)
FCTf3	46615612	37589608(80.64%)
FCTl1	52564414	40912264(77.83%)
FCTl2	46063810	35757220(77.63%)
FCTl3	42205750	32838820(77.81%)
FCTt1	49874712	39297964(78.79%)
FCTt2	54607384	42568446(77.95%)
FCTt3	52773394	41472038(78.59%)
FCTte1	46799570	36817424(78.67%)
FCTte2	45082254	35620418(79.01%)
FCTte3	47797284	37605278(78.68%)
FCTx1	53932124	44101118(81.77%)
FCTx2	56711888	46270594(81.59%)
FCTx3	55539344	45070232(81.15%)
MCTc1	45240904	36269316(80.17%)
MCTc2	44270454	35250956(79.63%)
MCTc3	43352584	34782328(80.23%)
MCTf1	39382930	32462026(82.43%)
MCTf2	49799480	41702126(83.74%)
MCTf3	53608262	44094472(82.25%)
MCTl1	41759122	32306986(77.37%)
MCTl2	52668864	41092678(78.02%)
MCTl3	41825858	32398618(77.46%)
MCTt1	49114000	38468044(78.32%)
MCTt2	42844744	34092386(79.57%)
MCTt3	49565828	39004940(78.69%)
MCTte1	44153602	33931496(76.85%)
MCTte2	51442562	39729462(77.23%)
MCTte3	45224394	34751238(76.84%)
MCTx1	56567570	45003570(79.56%)
MCTx2	51932996	40496860(77.98%)
MCTx3	52412860	41675238(79.51%)

A

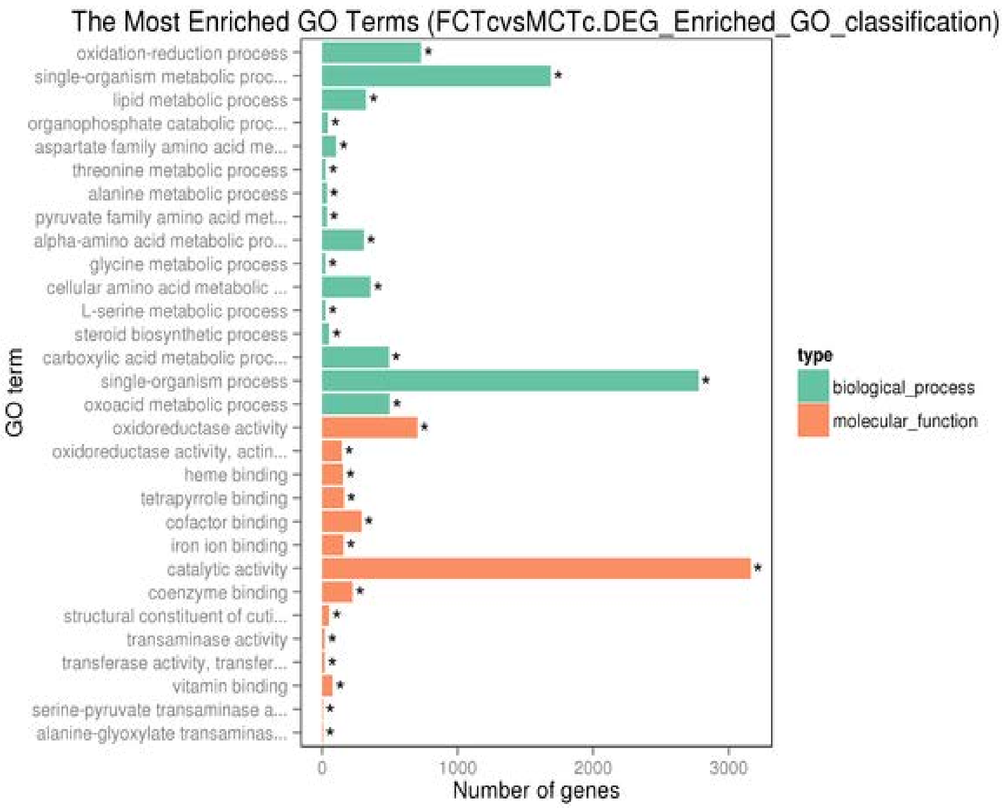

The Most Enriched GO Terms (FCTcvsMCTc.DEG_Enriched_GO_classification)
oxidation-reduction process
single-organism metabolic proc...
lipid metabolic process
organophosphate catabolic proc...
aspartate family amino acid me...
threonine metabolic process
alanine metabolic process
pyruvate family amino acid met...
alpha-amino acid metabolic pro...
glycine metabolic process
cellular amino acid metabolic ...
L-serine metabolic process
steroid biosynthetic process
carboxylic acid metabolic proc...
single-organism process
oxoacid metabolic process
oxidoreductase activity
oxidoreductase activity, actin...
heme binding
tetrapyrrole binding
cofactor binding
iron ion binding
catalytic activity
coenzyme binding
structural constituent of cuti...
transaminase activity
transferase activity, transfer...
vitamin binding
serine-pyruvate transaminase a...
alanine-glyoxylate transaminas...
GO term
0
1000
2000
3000
Number of genes
type
biological_process
molecular_function

B

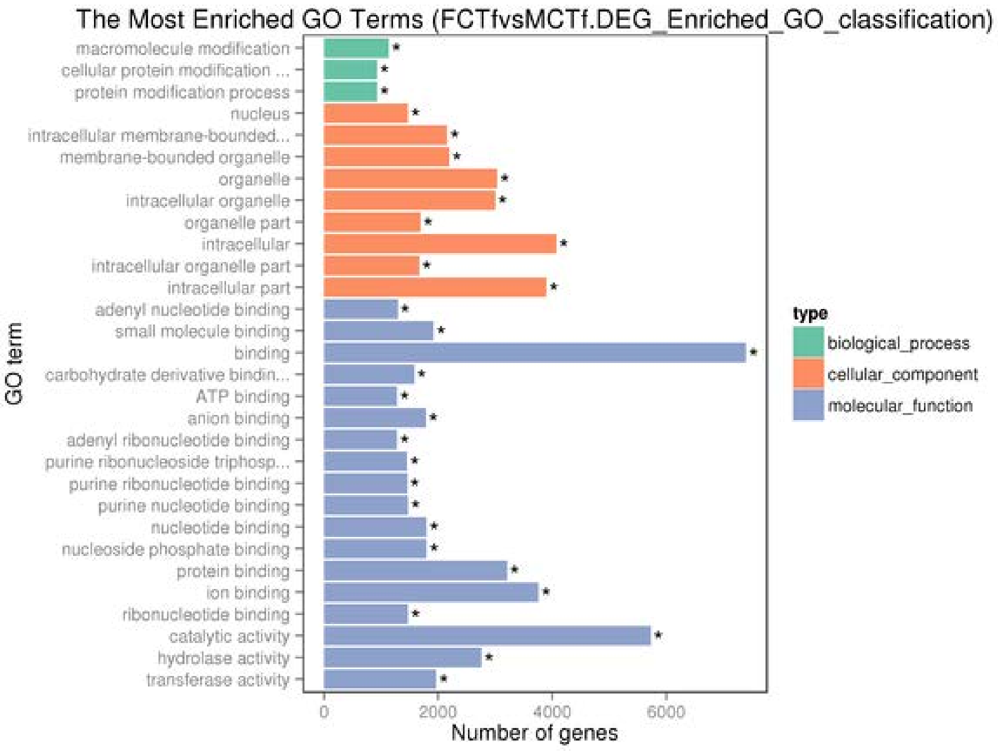

The Most Enriched GO Terms (FCTfvsMCTf.DEG_Enriched_GO_classification)
macromolecule modification
cellular protein modification ...
protein modification process
nucleus
intracellular membrane-bounded...
membrane-bounded organelle
organelle
intracellular organelle
organelle part
intracellular
intracellular organelle part
intracellular part
adenyl nucleotide binding
small molecule binding
binding
carbohydrate derivative bindin...
ATP binding
anion binding
adenyl ribonucleotide binding
purine ribonucleoside triphosp...
purine ribonucleotide binding
purine nucleotide binding
nucleotide binding
nucleoside phosphate binding
protein binding
ion binding
ribonucleotide binding
catalytic activity
hydrolase activity
transferase activity
GO term
0
2000
4000
6000
Number of genes
type
biological_process
cellular_component
molecular_function

C

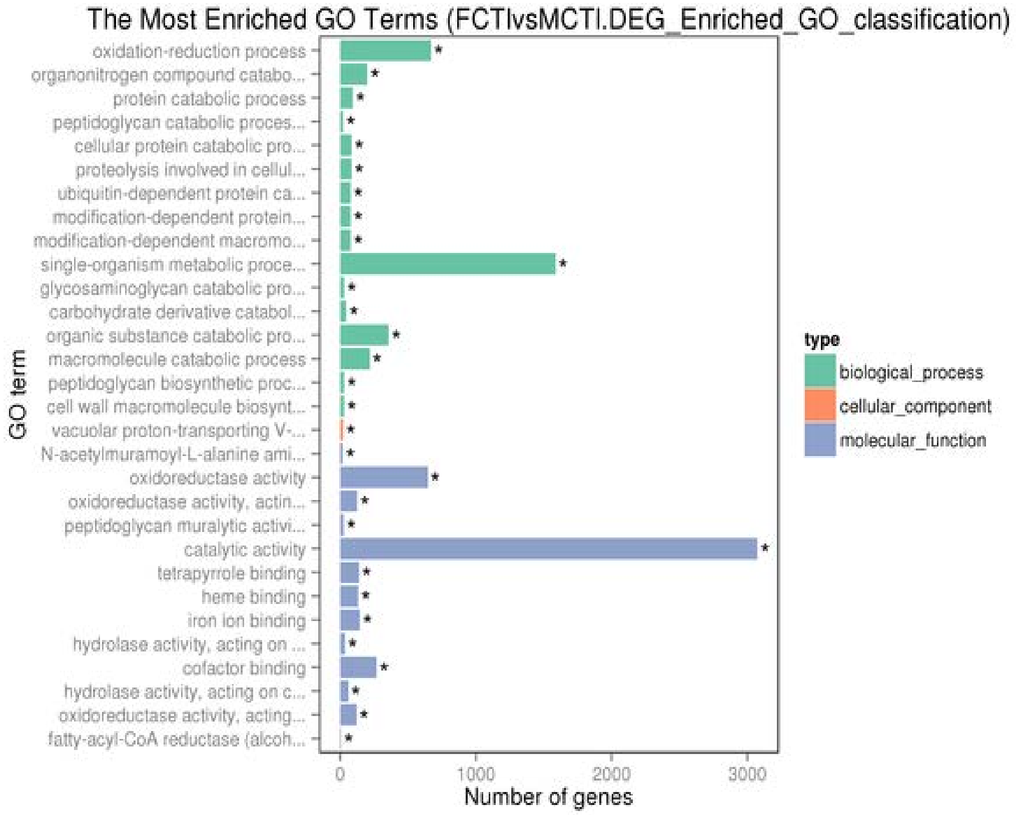
The Most Enriched GO Terms (FCTlvsMCTl.DEG_Enriched_GO_classification)
oxidation-reduction process
organonitrogen compound catabo...
protein catabolic process
peptidoglycan catabolic proces...
cellular protein catabolic pro...
proteolysis involved in cellul...
ubiquitin-dependent protein ca...
modification-dependent protein...
modification-dependent macromo...
single-organism metabolic proce...
glycosaminoglycan catabolic pro...
carbohydrate derivative catabol...
organic substance catabolic pro...
macromolecule catabolic process
peptidoglycan biosynthetic proc...
cell wall macromolecule biosynt...
vacuolar proton-transporting V-...
N-acetylmuramoyl-L-alanine ami...
oxidoreductase activity
oxidoreductase activity, actin...
peptidoglycan muralytic activi...
catalytic activity
tetrapyrrole binding
heme binding
iron ion binding
hydrolase activity, acting on ...
cofactor binding
hydrolase activity, acting on c...
oxidoreductase activity, acting...
fatty-acyl-CoA reductase (alcoh...
GO term
0
1000
2000
3000
Number of genes
type
biological_process
cellular_component
molecular_function

D

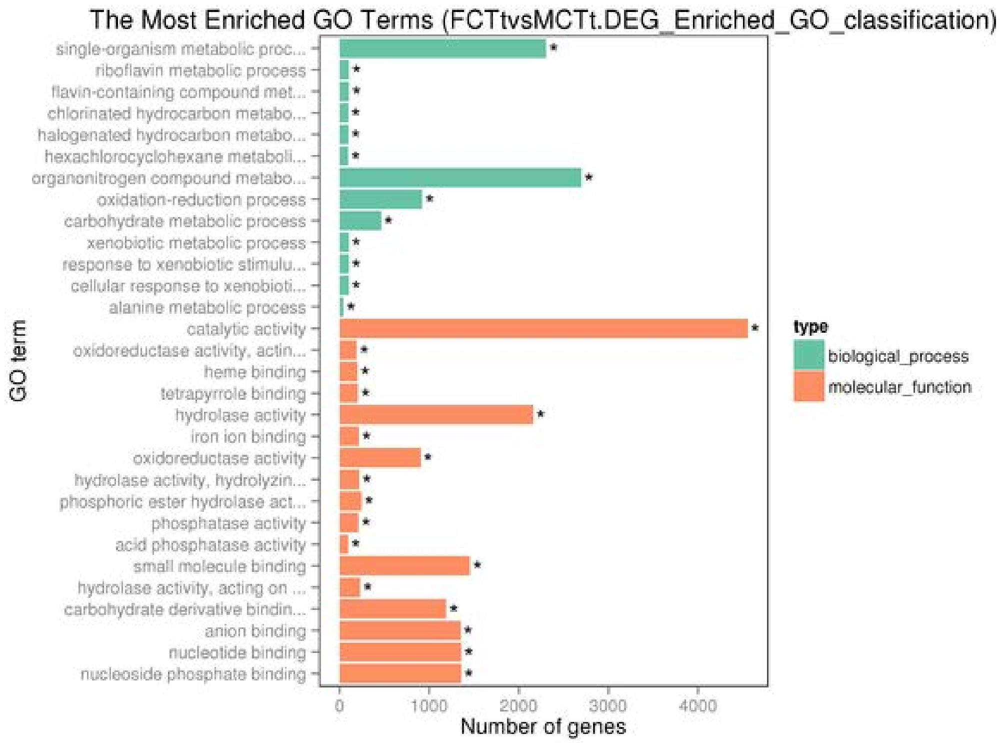
The Most Enriched GO Terms (FCTtvsMCTt.DEG_Enriched_GO_classification)
single-organism metabolic proc...
riboflavin metabolic process
flavin-containing compound met...
chlorinated hydrocarbon metabo...
halogenated hydrocarbon metabo...
hexachlorocyclohexane metaboli...
organonitrogen compound metabo...
oxidation-reduction process
carbohydrate metabolic process
xenobiotic metabolic process
response to xenobiotic stimulu...
cellular response to xenobioti...
alanine metabolic process
catalytic activity
oxidoreductase activity, actin...
heme binding
tetrapyrrole binding
hydrolase activity
iron ion binding
oxidoreductase activity
hydrolase activity, hydrolyzin...
phosphoric ester hydrolase act...
phosphatase activity
acid phosphatase activity
small molecule binding
hydrolase activity, acting on ...
carbohydrate derivative bindin...
anion binding
nucleotide binding
nucleoside phosphate binding
GO term
0
1000
2000
3000
4000
Number of genes
type
biological_process
molecular_function

E

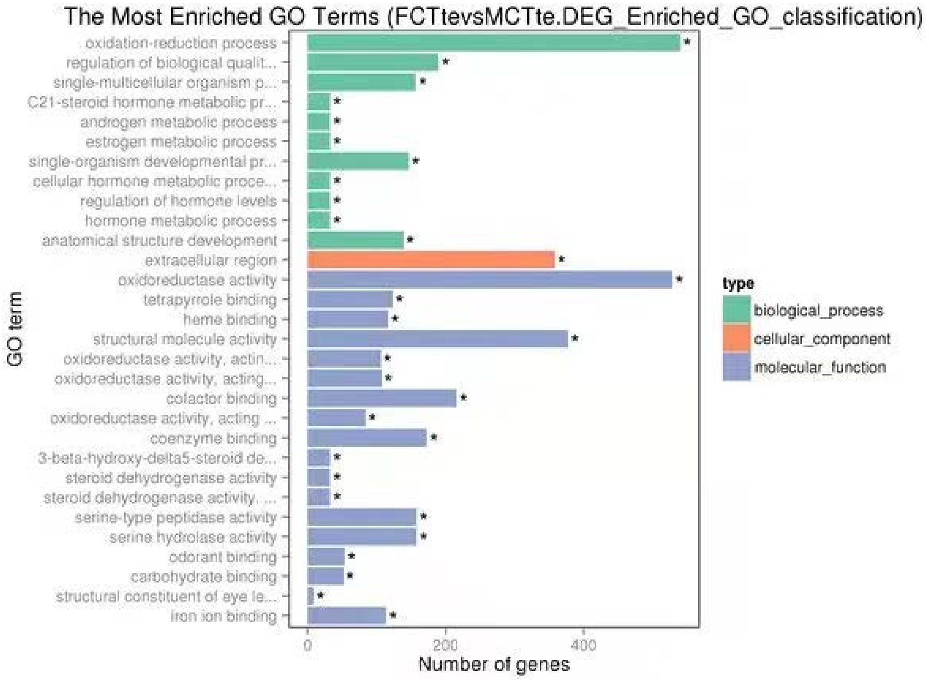

F

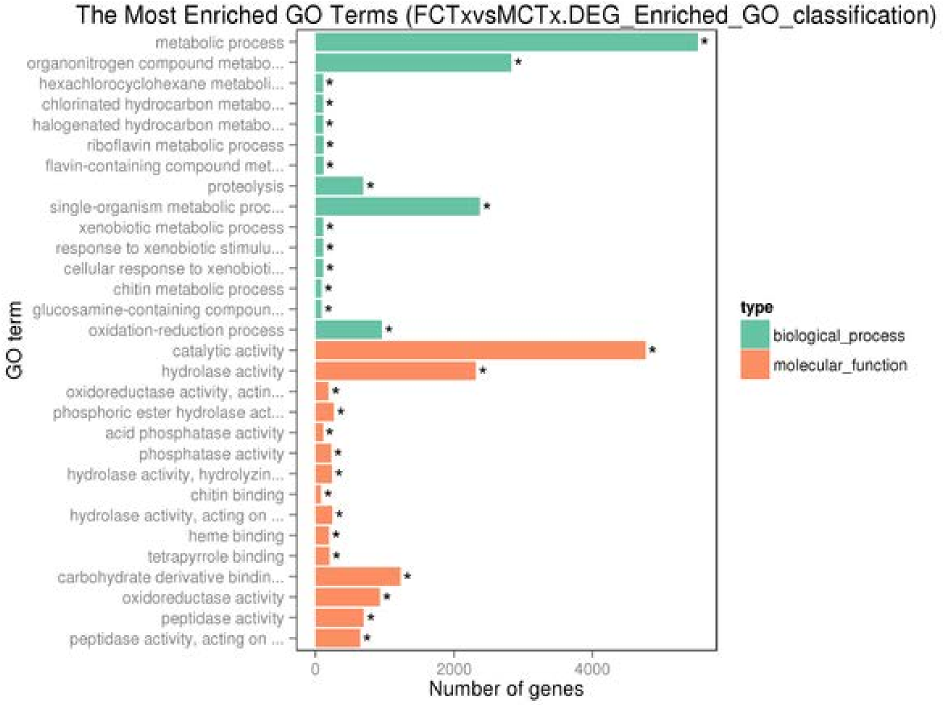

Figure S1. GO enrichment analysis of each sample between two groups (female *vs* male).

A (FCTc *vs* MCTc)

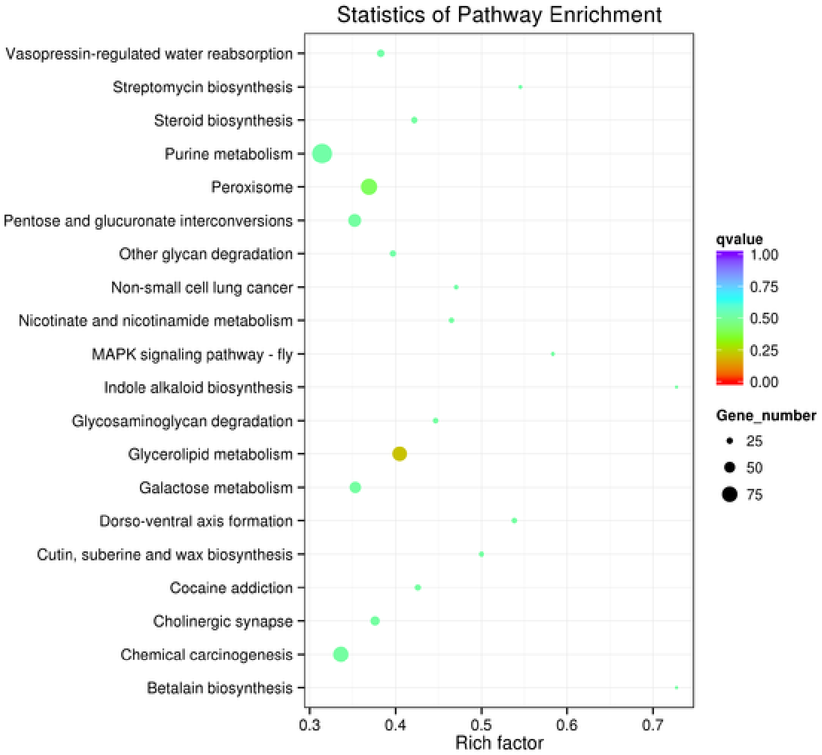

B (FCTf *vs* MCTf)

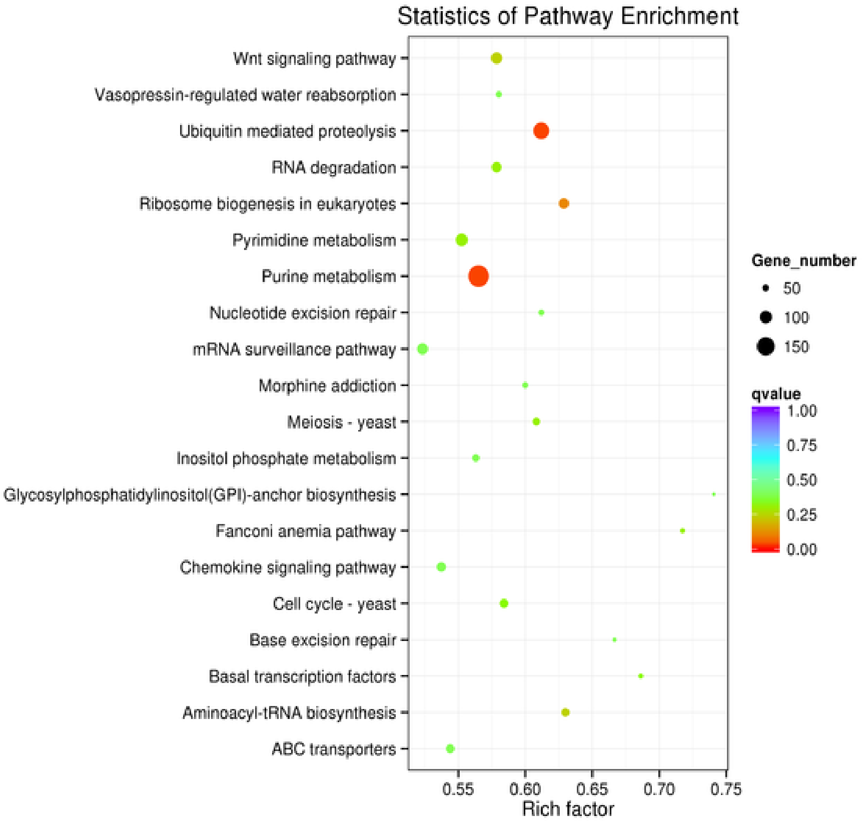

C (FCTl *vs* MCTl)

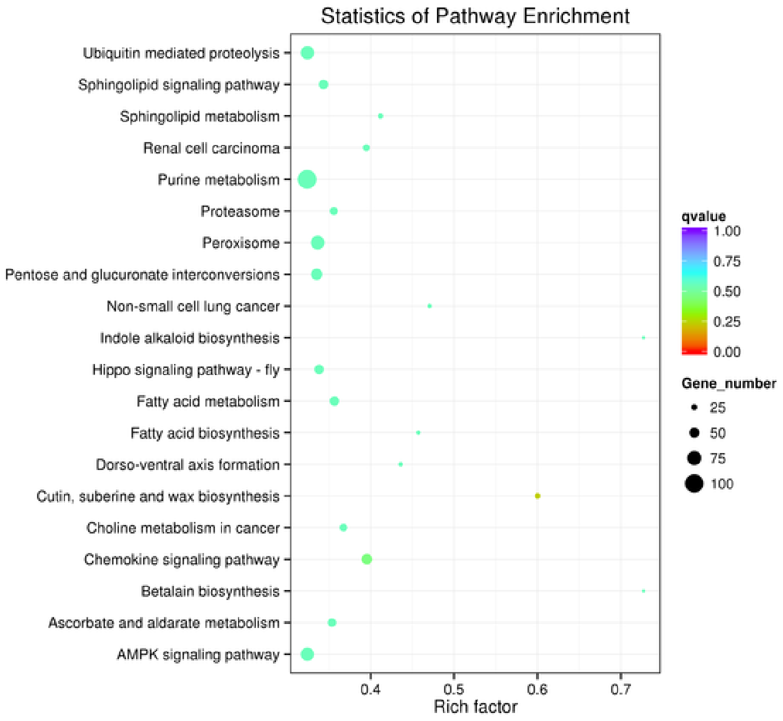

D (FCTt *vs* MCTt)

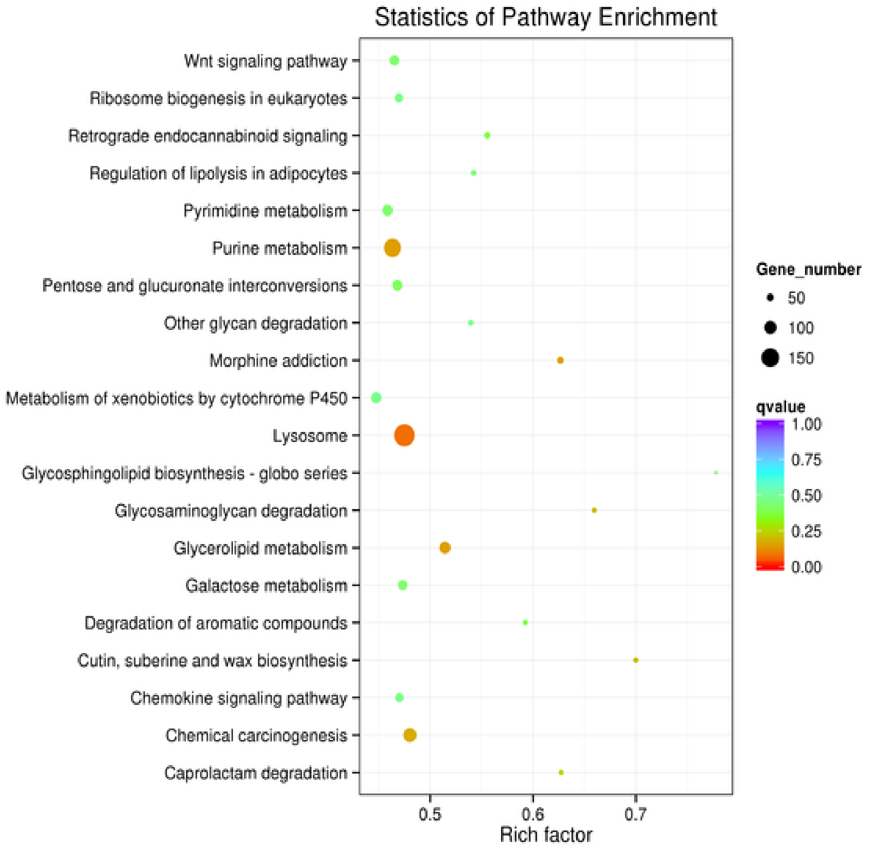

E (FCTte *vs* MCTte)

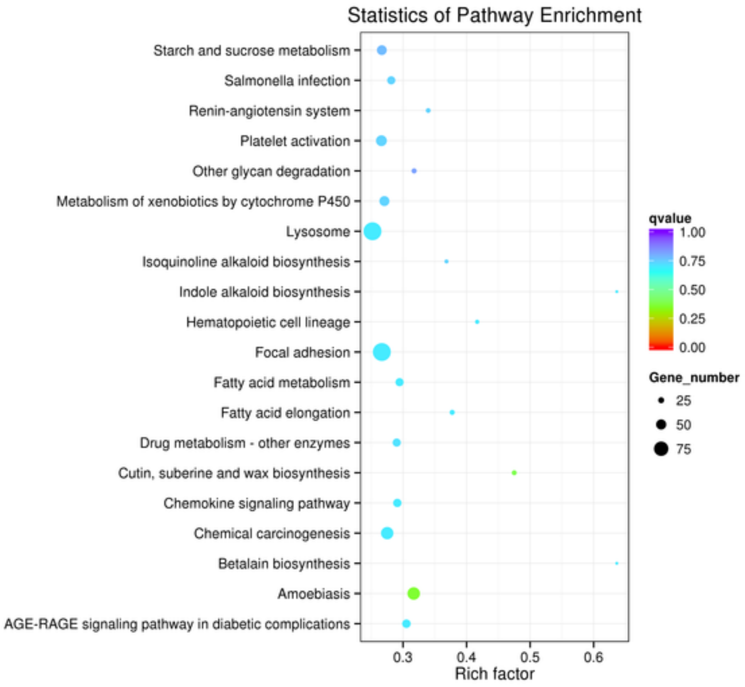

F (FCTx *vs* MCTx)

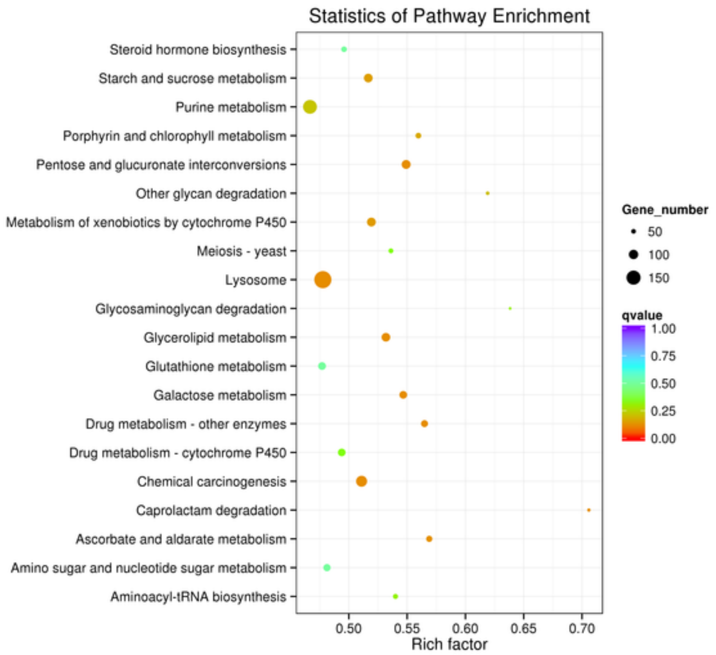

Figure S2. The statistics of 20 top KEGG pathways of DEGs.

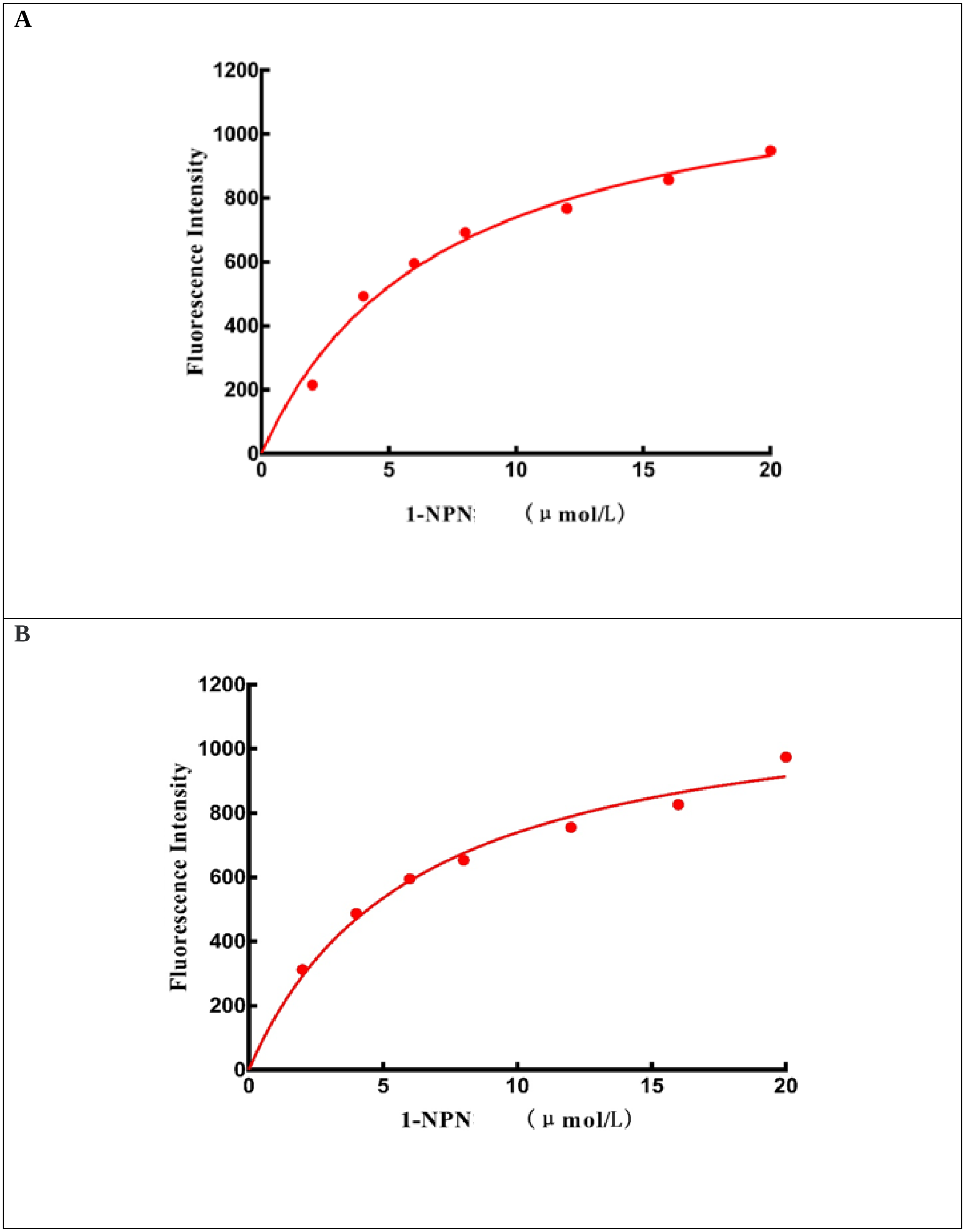

Figure S3. The binding curves of **(A)** of CcreOBP6 and 1-NPN **(B)** CcreOBP10 and 1-NPN.
